# Whole-Genome Sequence of *Lachnospiraceae* Bacterium NBRC 112778, Isolated from a Mesophilic Methanogenic Bioreactor

**DOI:** 10.1128/MRA.01412-19

**Published:** 2020-02-20

**Authors:** Kohji Morimura, Kenji Ueda

**Affiliations:** a Life Science Research Center, College of Bioresource Sciences, Nihon University, Fujiawa, Kanagawa, Japan; University of Southern California

## Abstract

Here, we report the draft genome sequence of Lachinospiraceae bacterium NBRC 112778. This anaerobic bacterium, isolated from a sweet potato-digesting mesophilic methanogenic bioreactor, represents a new genus related to the genus Anaerosporobacter. The 4.52-Mb circular genome harbored many genes involved in carbohydrate utilization, suggesting its adaptation to a saccharolytic environment.

## ANNOUNCEMENT

*Lachinospiraceae* bacterium NBRC 112778 was obtained from a mesophilic methanogenic bioreactor digesting sweet potato. The methanogenic sludge was inoculated onto reinforced clostridial medium (RCM) (1) containing 0.1% red starch (Megazyme) and 2% agar and anaerobically incubated at 37°C for 3 days in an AnaeroPack pouch (Mitsubishi Gas Chemical, Tokyo, Japan). An amylase-positive colony developed on the RCM plate forming a halo was purified by successive cultivation to yield the culture of NBRC 112778. 16S rRNA gene sequence analysis performed as previously described (2) indicated that the bacterium was most closely related to Anaerosporobacter mobilis (GenBank accession no. AY534872) (3). However, the low sequence identity (94.0%) suggested its affiliation within a new genus of the family Lachnospiraceae.

For genomic DNA isolation, NBRC 112778 was anaerobically cultured in a 300-ml Erlenmeyer flask containing 250 ml of RCM containing 0.1% soluble starch inoculated with the bacterial cells and incubated without shaking at 37°C for 24 h.

Genomic DNA was isolated from 0.4 g (wet weight) of cells obtained from ca. 1.5 liters of culture using an Isoplant II genomic extraction kit (Nippon Gene, Tokyo, Japan), and its quality was assessed by using a NanoDrop spectrophotometer (Thermo Scientific) and a Qubit fluorometer (Invitrogen). DNA was then sheared to an average size of 10 kb using g-TUBEs (Covaris). The genomic library was constructed using DNA template prep kit 1.0 and DNA/polymerase binding kit P6 (Pacific Biosciences [PacBio]). Size selection and quality analysis of the genomic library were performed using a BluePippin system (Nippon Genetics, Tokyo, Japan) and a 2100 Bioanalyzer (Agilent), respectively. A PacBio RS II sequencer was used to sequence the 10-kb library of the NBRC 112778 genome using P6-C4 chemistry. For the following analysis, default parameters were used for all software.

Quality control of PacBio readings was performed using Hierarchical Genome Assembly Process v2 (HGAP2) implemented with SMRT Analysis software v2.3.0 (PacBio) (4). *De novo* assembly for 112,408 PacBio subreads (*N*_50_, 10,478 bp) was performed successfully to closure by HGAP2, yielding a single contig. The assembled genome data were circularized and subjected to annotation using the Rapid Annotations using Subsystems Technology (RAST) server (5). The result of annotation was edited to fit the DDBJ format and then submitted.

A single contig generated from the assembly consisted of 4,521,945 bp with a G+C content of 36.6%. The sequence assembly did not contain any plasmid. RAST predicted 4,174 genes for protein-coding sequences, 24 rRNA genes, and 69 tRNA genes. A whole-genome survey using the SEED Viewer (6) showed that NBRC 112778 harbored many genes for (oligo)saccharide utilization, including 17 genes for utilizing maltose and maltodextrin and 7 for utilizing lactose and galactose. This genomic information implies that NBRC 112778 is a carbohydrate degrader when present in a saccharolytic environment.

### Data availability.

The conditions for the isolation and cultivation of NBRC 112778 are available in the BioSample database (accession no. SAMD00112399). The 16S rRNA gene sequence and complete genome sequence of NBRC 112778 have been deposited in DDBJ/GenBank under accession no. LC330916 and AP018794, respectively. We submitted the raw sequencing reads to DRA under accession no. DRR198068.
